# Enhancing AI Research for Breast Cancer: A Comprehensive Review of Tumor-Infiltrating Lymphocyte Datasets

**DOI:** 10.1007/s10278-024-01043-8

**Published:** 2024-05-28

**Authors:** Alessio Fiorin, Carlos López Pablo, Marylène Lejeune, Ameer Hamza Siraj, Vincenzo Della Mea

**Affiliations:** 1 Oncological Pathology and Bioinformatics Research Group, Institut d’Investigació Sanitària Pere Virgili (IISPV), C/Esplanetes no 14, 43500 Tortosa, Spain; 2 Department of Pathology, Hospital de Tortosa Verge de la Cinta (HTVC), Institut Català de la Salut (ICS), C/Esplanetes no 14, 43500 Tortosa, Spain; 3 Department of Computer Engineering and Mathematics, Universitat Rovira i Virgili (URV), Tarragona, Spain; 4 Department of Mathematics, Computer Science and Physics, University of Udine, Udine, Italy

**Keywords:** TIL, Datasets, Breast Cancer, Immunology, Deep Learning, Computer Vision

## Abstract

The field of immunology is fundamental to our understanding of the intricate dynamics of the tumor microenvironment. In particular, tumor-infiltrating lymphocyte (TIL) assessment emerges as essential aspect in breast cancer cases. To gain comprehensive insights, the quantification of TILs through computer-assisted pathology (CAP) tools has become a prominent approach, employing advanced artificial intelligence models based on deep learning techniques. The successful recognition of TILs requires the models to be trained, a process that demands access to annotated datasets. Unfortunately, this task is hampered not only by the scarcity of such datasets, but also by the time-consuming nature of the annotation phase required to create them. Our review endeavors to examine publicly accessible datasets pertaining to the TIL domain and thereby become a valuable resource for the TIL community. The overall aim of the present review is thus to make it easier to train and validate current and upcoming CAP tools for TIL assessment by inspecting and evaluating existing publicly available online datasets.

## Introduction

Breast cancer (BC) is the most common cancer in women and is a significant threat to public health worldwide [1]. The role of immune cells in the primary tumor in BC disease progression cannot be underestimated since, by modeling the immunological context, they reflect the immune response determined at the time of diagnosis [2, 3]. The ability to estimate the prognostic value of immune cells has been refined further with the rise of more reliable methods to determine immune cell functional status and phenotypes. The findings demonstrate that immune cells in BC can affect cancer cell behavior and that the malignant disease arises [4], in part, by a bilateral relation between cancer cells and their immunological microenvironment [5–8].

Lymphocytes have been identified as one of the immune cells with the strongest prognostic implications of the various forms of immune populations [9]. Higher concentrations of tumor-infiltrating lymphocytes (TILs), lymphocytes that migrate into the tumor microenvironment, have been associated with better prognosis and treatment response in BC, especially in two subtypes: HER2-positive and triple-negative breast cancer (TNBC) [10, 11].

An initial finding of Aaltomaa et al. has prompted significant and ongoing TIL assessment and research [12, 13], leading to the establishment of the TIL Working Group (TIL-WG), an international group of experts whose main objective is to update TIL assessment guidelines and, in particular, to standardize Visual TIL Assessment (VTA) [14]. The TIL-WG strives to minimize the subjectivity in evaluation that arises from the high level of inter-observer variation between specialists [2, 15–17].

By studying the spatial distribution and quantity of TILs, assessed by a histopathological examination using hematoxylin and eosin (H&E)-stained images [2], experts can gain valuable insights into patients’ prognosis and response to treatment [18].

For a comprehensive understanding of the immune landscape of BC, we can also take into consideration immunohistochemistry (IHC)-stained images of biomarkers related to TILs, like CD4, CD8, FOXP3, CD20, and CD22 biomarkers [19–23]. The first three of these highlight some subclasses of a type of immune cell, the T lymphocyte, that appears in the presence of infected or cancerous cells [24]. The latter two highlight B lymphocytes [25], which are also evaluated as TILs in H&E-stained images. Note that only H&E-stained images are used to evaluate TILs in clinical practice, whereas IHC-stained images are still used for research purposes only [2].

The evaluation of biomarkers related to TILs in H&E or IHC-stained images has shown significant potential when based on advanced artificial intelligence (AI) techniques, such as deep learning (DL) [26–28]. These methods have the potential to yield promising results that overcome the problem of inter-observer variation, but more data and more parameters need to be incorporated to create more accurate and powerful models.

DL-based approaches in BC research should be trustworthy and reliable, so efforts should be made to collect a variety of representative datasets that are free from bias and to verify the consistency of the annotations within the datasets to minimize errors made during the annotation process by introducing data quality-control procedures. An example of such a procedure is the use of relations verification defined in a spatial logic by applying Discrete Mereotopology techniques to Mathematical Morphology [29, 30].

To reinforce the value of TILs, we will first address the importance of the immune response, which plays a significant part in cancer evolution. We will describe the assessment of TILs using histological image analysis employing digital pathology, a promising and growing area of research in the field of immune responses. We will then discuss the development of computer-assisted pathology (CAP) tools, which require massive amounts of data to train models using techniques involving advanced AI algorithms. In addition, we will suggest ways to make the TIL annotation process less time-consuming in future datasets, while maintaining its ground-truthness. At this point, we will highlight the primary aim of our review, which is to provide a perspective on the datasets used for TIL assessment, in particular those of publicly available histological images, to see whether the TIL research works with the same batch of data. To broaden the spectrum of the datasets and to allow the models to be more widely generalizable, we will also look for more available data on TILs in other tumors. For each dataset, we will then evaluate the main approaches for assessing TILs based on the types of annotations provided. Thereafter, we will discuss the ideas about the datasets that could contribute most substantively to the research by filling in gaps or addressing limitations evident in the current literature.

## TIL Assessment for Evaluating Cancer Progression

TILs are predictive prognostic markers in BC because they provide a snapshot of the tumor scenario and are one of the best examples of the association between natural defenses and carcinogenesis [31]. Thus, we can conceive of TILs as an unloaded weapon whose drug-induced reactivation can lead to the restoration of formerly fully operational natural anti-cancer defenses [31].

TILs are essential for analyzing the immunological environment of BC and other malignancies like colorectal cancer or other kinds of solid tumors [17, 32, 33].

TILs should be frequently evaluated as novel prognostic and therapy-predicting markers, particularly in the most aggressive breast lesions, such as the triple-negative and HER2-positive molecular subvariants [31, 34]. TILs have been studied independently of immune blockade agents as prognostic indicators influencing BC outcomes in chemotherapy trials in several publications [31, 35, 36].

However, TIL assessment has not featured in pathological reports even when it is accepted as a prognostic factor, as stated in the St. Gallen 2023 guidelines [37]. The role of TILs in treatment decisions remains unclear because the data on TILs are still considered insufficient to enable a reliable choice of specific therapy regimens to be made and to decide whether to withhold treatment [31, 38]. As a result, currently clinicians are not recommended to base their therapy decisions solely on TILs [38]. For this reason, TILs should not be treated as an independent variable [39, 40], but rather interpreted in conjunction with other prognostic variables like tumor and lymph node status to provide clinicians with all the prognostic information they need to examine treatment options reliably with their patients [38].

In the near future, TIL research will be able to guarantee a novel standardization of TIL assessment by improving on the approximate semiquantitative evaluation that is currently practiced, which is affected by a substantial degree of inter-observer variation [2, 14].

## Enhancing Diagnosis Through Computer-Assisted Pathology

One of the primary advantages of digital slides over traditional glass slides is the ability to apply quantitative automatic image analysis algorithms with the introduction of AI techniques, leading to the creation of computer-assisted pathology (CAP) tools [41].

By using these instruments, it is possible to reduce inter-observer error and subjectivity of pathologists and thereby help them with the assessment process [14].

CAP tools, with AI integration, offer support in a range of tasks related to computer vision; “Computer Vision Tasks in CAP Tools” section describes possible applications. However, to fully exploit the power of AI in CAP tools, large-scale annotated datasets are indispensable. Given the time-consuming nature of dataset creation, in “Optimize the Annotation Phase Time” section, we will delve into methods to expedite and enhance this process.

### Computer Vision Tasks in CAP Tools

Given the advances in computer vision and AI, CAP tools are becoming increasingly important in digital pathology tasks [41], such as automatic tissue segmentation and nucleus detection [42–44]. Even though certain CAP tools can quantify specific nuclei, such as TILs, they do so with varying degrees of difficultly. By considering TILs, a classification strategy can determine whether there are TILs present in a given image. By employing the localization approach, it is possible to specify the regions where TILs are located, like box shapes [45].

This strategy can be beneficial to weakly supervised learning, in which a whole slide image (WSI) that we claim contains TILs can be split into tiles and the locations of the TILs then checked [46]. By so doing, we can gain an approximate idea of where the TILs are in terms of spatial localization, even if this is not sufficient to allow them to be quantified [47].

Another helpful method for quantification that is more complex than combining classification and localization involves drawing a box around each TIL and counting the frequency of boxes [48, 49]. A variant of the method consists of placing a point over the object instead of a bounding box. This is useful when, for example, there are multiple small objects (as is the case in TIL detection), since under such circumstances box usage would cause too much confusion and, due to the overlap of many boxes, would give unclear results [50].

The next stage is semantic segmentation, which involves drawing a boundary around each object and determining the pixel-level features. Semantic segmentation entails labeling every pixel in an image and determining the class to which it belongs [51]. It is feasible to define which pixels are not part of a TIL in this manner [52, 53]. However, if there are more nearby TILs, we will not be able to estimate their frequency accurately, but instead, we will see a region with TILs [48]. For example, the segmentation mask task can be beneficial for identifying tissue regions, so objectives are not directly related to quantification. Nevertheless, it can be of practical value to distinguish between stromal and intratumoral TILs, which is essential for the obtaining a TIL score in BC [2, 14].

Finally, there is a process, known as instance segmentation, that advances semantic segmentation. Rather than giving all objects in a class equal pixel values, this process aims to segment and display various instances of the same class [54]. By doing so, we may establish more exact boundaries for semantic segmentation and object identification, by which means we can determine the number of objects [55–57].

### Optimize the Annotation Phase Time

To effectively train CAP tools, it is essential to annotate WSIs. Pathologists typically begin this process by manually annotating a limited number of WSIs [58]. These annotations serve as the initial labeled data for CAP tool training [59, 60]. Once this first step has been completed, the remaining WSIs can be annotated using a semi-supervised learning approach, which reduces the amount of manual annotation by allowing pathologists to intervene only to ensure the accuracy and refinement of the generated annotations [61, 62].

With the advent of DL techniques, there has been an increase in demand for many annotations [61]. DL models have a considerable capacity to learn detailed patterns and features from data due to their complex architectures. While DL models require a large amount of training data, they have the potential to yield outstanding performance levels.

As a result, having a large dataset with numerous annotations becomes critical if the promise of DL in pathology is to be fully realized [63].

Collecting these data can be time-consuming, particularly when manual annotations, which can also become monotonous and repetitive, are involved. For these reasons, there is an urgent need to identify novel techniques to improve the stage of dataset creation [58, 61, 64].

We may discover smart approaches to simplifying the process by carefully reviewing the annotation methods used in some of the datasets mentioned hereafter.

First, we note that a common approach to achieving large numbers of annotations consists simply of having more people making the annotations. Since it is difficult to find several expert pathologists who are available for the task, structured crowdsourcing is a possible approach, whereby people with less expertise make annotations in accordance with their level of skills, and their work is mentored and eventually corrected by expert pathologists [65, 66].

The review phase consists of correcting and giving an overlay of the segmentation. In [65], the review phase was mainly exploited for annotating non-predominant or challenging classes. Production of the latest version of this dataset, which is mentioned in the work of Amgad et al., involved an intriguing new technique that includes non-pathologist nucleus labels [64]. Two main approaches were employed in this work: one focused on breadth, gathering single-rater annotations over many fields of view (FOVs) to obtain the majority of the data in the study, while the other assessed interrater reliability and agreement by gathering annotations from numerous non-pathologists for a smaller selection of common FOVs. Pathologists also provided annotations for these FOVs to assess non-pathologists’ reliability [64].

To lower the labeling burden, the method of initial labeling followed by a review by professional pathologists is employed. However, ensuring the accuracy of labeling by non-pathologists remains a challenge. The re-examination process is still time-consuming and labor-intensive if the initial annotation is not of high quality, and it requires the involvement of multiple specialists to prevent subjective errors [64].

Amgad et al. also introduced an algorithmic recommendation for nucleus boundaries and classes that provides instructions to annotate other nuclei with bounding boxes by clicking on nuclei with correct border recommendations [64].

Adding an automatic proposal, made, for instance, by a DL model, provides an iterative learning strategy whereby each iteration produces better annotation suggestions that require less manual adjustment [61, 67].

We suggest further approaches to the introduction of annotations by considering different people with variable levels of expertise, allowing non-experts to carry out the main tasks at their level of skill under the supervision of expert pathologists. Tools like MONAI [68, 69] and Quick Annotator [58] are available to make this manual adjustment and thereby facilitate better automatic annotation proposals. These tools, which use an active learning framework for continuous learning, can be integrated into digital pathology and WSI analysis platforms like QuPath [70].

Using these tools makes it possible to take advantage of the efficiency of weak labeling methods, which need substantially less time and resources. We can extract further annotations from unlabeled images by starting with a small set of annotations made by domain experts [47]. This idea of generating annotations from unlabeled data was previously investigated in traditional CAP tools, which used image-processing-based approaches like thresholding to extract annotations [71]. These rule-based techniques, however, are extremely task-specific and require domain expertise for troubleshooting and optimization.

## Comparative Analysis of Datasets for TIL Research

This section aims to provide an overview of the publicly available datasets for TIL assessment using H&E images (Table 1). On Table 1, detailed information such as magnifications and size of the datasets is presented, enhancing our understanding of their composition and potential usability.

**Table 1:** Public and available datasets for TIL assessment

**Dataset Name**	**Year**	**Annotations**	**Number of images**	**Origin**	**Scanner**	**Image magnification**	**Task**	**Cancer types**	**Annotation Type (level)**	**License**
Janowczyk [75]	2016	1,735 ROIs and 3,064 lymphocyte centers	42 1,000×1,000 ROIs and 100 100×100 ROIs	CWRUC	Not reported	20×/40×	Epithelial segmentation and TIL detection	Breast	Pixel	CC Attribution-NonCommercial-ShareAlike 3.0
CRCHisto [76]	2016	6,971 inflammatory nuclei centers	100 images of 500×500 pixels	UHCW	Omnyx VL120 scanner	20×	Inflammatory cell detection	Colorectal	Pixel	Attribution-NonCommercial-ShareAlike 4.0 International
TNBC [77]	2017	Numbers of inflammatory cells and nuclei annotations not reported	Three to eight 512×512 patches for 11 patients (from 50 WSIs) / 1,000×1,000 patch for each of the 30 WSIs	DS1: Curie / DS2: IITG	DS1: Philips Ultra Fast Scanner 1.6RA/ DS2: Aperio	40×/40×	Inflammatory cell instances	DS1: breast/ DS2: 7 organs	Pixel	CC Attribution 4.0 International
TIL-WSI-TCGA [78]	2018	Number of annotations not reported	3,769 WSI split into 100×100 squares	TCGA	Aperio	20×	TIL patch-level assessment	13 tumor types	Patch	TCIA Data Usage Policy and the CC Attribution 3.0 Unported License
CPM-17 [79]	2019	Number of lymphocyte annotations not reported	32 tile images 500$\times$500 to 600$\times$600	TCGA	Aperio	20× and 40×	Lymphocyte segmentation	NSLC, HNSCC, GBM, LGG	Pixel	Not mentioned
CoNSeP [80]	2019	1,317 lymphocytes, 332 plasma cells, 30 neutrophils, 52 eosinophils annotated	41 tiles 1,000×1000	UHCW	Omnyx VL120 scanner	40×	Inflammatory cell instance segmentation	Colorectal	Pixel	Apache 2.0 license
BCSS [65]	2019	20,340 annotated tissue compartments over 151 ROIs (one ROI per WSI)	151 WSIs	TCGA	Aperio	40×	Tissue compartments segmentation	Breast	Pixel	CC0 1.0 Universal (CC0 1.0) license
MoNuSAC [81]	2020	23,460 labeled lymphocytes	Pre-selected nucleus-rich regions of 71 WSIs	TCGA	Aperio	40×	Lymphocyte instance segmentation	Breast, kidney, liver, and prostate	Pixel	CC BY-NC-SA 4.0
PanNuke [82]	2020	3,592 lymphocytes, 1,465 plasma cells, 61 neutrophils, 37 eosinophils annotated	96 WSIs from 54 patients	From Kumar et al. [83], CPM-17, 15 ROIs from TCGA manually labeled ROIs of bone marrow	Aperio	20×/40×	Inflammatory cell instance segmentation	19 tumor types	Pixel	Attribution-NonCommercial-ShareAlike 4.0 International
Lizard [84]	2021	101,413 lymphocytes, 28,466 plasma cells, 4,824 neutrophils, 3,604 eosinophils annotated	91 colon image regions with an average size of 1,016×917 pixels	GlaS [85], CraG [86], CoNSeP, TCGA and DigestPath [87]	Zeiss MIRAX MIDI Slide Scanner,Omnyx VL120 scanner, Aperio, KFBIO FK-Pro-120 slide scanner	20×	Colonic nuclear instance segmentation and classification	Colon	Pixel	Attribution-NonCommercial-ShareAlike 4.0 International
NuCLS [64]	2021	32k lymphocytes, 13k plasma cells, 2k macrophages, 109 neutrophils, 4 eosinophils annotated	A ROI from each of the 125 WSIs	TCGA	Aperio	40×	sTIL detection and sTIL segmentation	Breast	Pixel	CC0 1.0 Universal (CC0 1.0)
Post-NAT-BRCA [88]	2022	Number of annotations not reported	96 WSIs from 54 patients	SHSC	Aperio	20×	TIL segmentation and cellularity degree compartment segmentation	Breast	Pixel	TCIA Data Usage Policy and the CC Attribution 3.0 Unported License
Abousamra [46, 89]	2022	304,097 image-annotated patches	Patches of size 100$\times$100	TCGA	Aperio	20×	TIL patch-level assessment	Pancancer	Patch	CC Attribution 4.0 International
CoNIC [90, 91]	2022	Lizard’s annotations [84] + internal 39,884 nuclei annotations	Patches of size 256$\times$256	Lizard + internal colon biopsy dataset	Zeiss MIRAX MIDI Slide Scanner,Omnyx VL120 scanner, Aperio, KFBIO FK-Pro-120 slide scanner	20×	sTIL detection and sTIL segmentation + nucleus detection	Colon + internal colon biopsy	Pixel	Attribution-NonCommercial-ShareAlike 4.0 International
TIGER [92] - WSIROIS	2022	30,524 sTILs + 20,340 annotated tissue compartments	Preselected-ROIs from 195 WSIs	BCSS and NuCLS datasets, RUMC and JB	Aperio	20×	Tissue compartment segmentation, sTIL detection	Breast	Pixel	CC BY-NC 4.0 license
TIGER [92] - WSITILS	2022	No manual annotations	82 WSIs	RUMC and JB	Aperio	20×	Tissue compartments segmentation, sTIL detection	Breast	WSI	CC BY-NC 4.0 license
PanopTILs [52]	2022	200k semi-automatic TIL annotations	210 WSIs (125 WSIs + 85 WSIs)	BCSS and NuCLS datasets, Cancer Prevention Study II cohort	Aperio	40×	sTIL detection and sTIL segmentation	Breast	Pixel	CC0 1.0 license

For a more complete perspective of the available datasets, we extend the search to encompass cancer types in addition to BC. We also examine datasets not based on TILs but also those for lymphocytes and inflammatory cells. TILs are specific kinds of lymphocytes, so we can gather additional information from datasets for lymphocyte evaluation, such as that on morphological features, for TIL assessment [72]. We then look for datasets that assess inflammatory cells because these can be relevant for TIL scoring when performed on round inflammatory cells, omitting polymorphonuclear cells solely in the intratumoral region in cutaneous melanoma [73]. As a result, we can use inflammatory cell datasets from different cancer types to generalize predictive models [74].

**Fig. 1:**
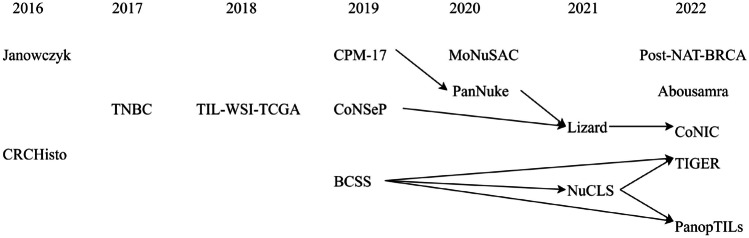
Dependencies in the TIL datasets described in Table 1. The years indicate when datasets were created; the arrows indicate the dependencies between them

As can be seen in Table 1, some datasets are a combination, or a selection of some parts, of other datasets. Figure 1 outlines the dependencies of the datasets described in Table 1.

We recognize that there are other online datasets, but access to them is either restricted or requires prior registration.

One important dataset that requires prior registration is the ATLAS of Histopathology database, a large-scale, patch-level annotation of different components of tissues created in 2019 by the Multimedia Lab of the University of Toronto. This dataset requires an End User License Agreement (EULA) to be accepted [93].

There are datasets for TIL assessment without annotation that feature only associated clinical data. One such example is that proposed by Shvetsov and coworkers, called UiT-TILs, that can be used to clinically validate TIL classifications [94]. The UiT-TILs dataset contains 1189 image patches from 87 non-small cell lung cancer (NSCLC) patients with matched clinical data, and it is a subset of another dataset, reported by Rakaee et al. [95]. A similar dataset was presented by Fassler’s team, in which they processed data not only from the TCGA, but also from the UNC CBCS Phase 3 cohort, which contains 2998 cases with 1138 diagnostic WSIs from representative blocks, and related follow-up recurrence and survival data [96, 97].

### Dataset Goals Over the Years

In the first part of “Comparative Analysis of Datasets for TIL Research” section, we analyzed the datasets for evaluating TILs, lymphocytes and inflammatory cells. This raises questions about whether the focus of these datasets has changed over time and what subjects and issues they target. In search of answers, we extracted the keywords of each abstract through ChatGPT APIs since this is a fast and intuitive method. We decided not to examine author-entered keywords to minimize potential bias and ensure universality since some dataset articles do not include author-entered keywords.

First, we extracted the texts of the abstracts from the papers relating to the datasets shown in Table 1. We then applied OpenAI APIs to them in order to ask ChatGPT to identify the main words in each paper’s abstract. We did this because we assumed that most of the important keywords are almost always mentioned in a paper’s abstract. Finally, we grouped the keywords from the dataset articles by year and carried out topic modeling for each year using a Latent Dirichlet Allocation (LDA) model [98], which enabled us to discover the hidden relationships in the keyword collection and ultimately the main topic for each year [99, 100].

**Fig. 2:**
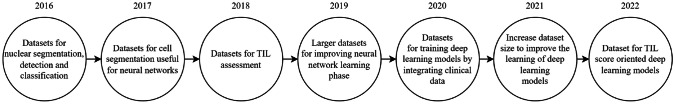
Change of topics over the years in the TIL assessment datasets analyzed

Figure 2 shows how the dataset goals change over time, as revealed by the analysis of the LDA model outcome. It is clear that the research community has continued to present ever-larger datasets over the years because the advent of DL has meant that we now need substantial quantities of data to train neural networks.

We note that the research community has invested significant resources and will continue to do so in order to make annotated datasets for training CAP tools. While these technologies use efficient and innovative approaches to save time, the process remains complex and demands effective communication among experts from diverse sectors [41].

The aforementioned trend shows that TIL evaluation is becoming more dependent on the contribution of CAP tools. This is crucial because it will progressively reduce the differences from expert assessments over time [14]. As a result, in the TIL evaluation scenario, we note a growing inclination to merge the experience of experts from different fields [2, 14].

## Conclusions and Proposals for Future Challenges

This review compares datasets used for TIL assessment and encompasses datasets about lymphocytes and inflammatory cells, since, as mentioned in “Comparative Analysis of Datasets for TIL Research” section, they are related to the TIL assessment scenario.

The research community is working to make larger datasets for TIL assessment and should also provide novel TIL datasets for different tumor types in addition to BC to enhance this area of investigation [92]. It is essential to include annotated images from various scanners in order to leverage models trained on these datasets effectively. This approach ensures improved generalization of the TIL assessment, making the models helpful across a variety of scanners rather than being limited to the performance of a specific one [101–103].

By adopting this strategy, CAP tools will become more valuable to experts and enable them to carry out TIL assessments of which they can have greater confidence.

As stated in “Introduction” section, TIL assessment is achieved using H&E images. Nevertheless, there is a supplementary and non-standardized method for measuring TILs, consisting of quantifying immune biomarkers of specific subpopulations of TILs such as CD4, CD8, FOXP3, CD20, and CD22 [19–23]. The information about the immune markers can provide us with more insights into TILs and about their distribution and spatial relationships, as shown by two studies [104, 105]. However, we require IHC images of them, such as sections from the paraffin block, to make this evaluation. It should be noted that the sections, even if very close (e.g., 4 $\mu$m [106]) and from the same paraffin block, can vary slightly [107]. There are also a large number of variables that influence antigen staining in paraffin-embedded tissues, such as the type of fixative, fixation time, tissue processing, the level of antigen expression and preservation, and also the clone and the dilution of the antibody used, the antigen-retrieval method, and the detection system and chromogen [108, 109]. Other procedures use multiplexed IHC images to apply diverse IHC staining in a single section. However, these are more expensive and can be beset with problems of antibody compatibility and tissue penetration [110].

To broaden the scope of TIL assessment research, the research community should make these IHC images public and widely available. By doing so, or, better yet, by offering the ground truth, we can rise to exciting challenges like the one overcome to distinguish HER2-positive from HER2-negative BC specimens solely through the evaluation of H&E slides [111]. Thus, a novel aim should be to quantify immune markers directly on H&E-stained images.

## Data Availability

Public and available datasets supporting the findings of the review are available within the article.

## Abbreviations

AI: Artificial Intelligence
BC: Breast cancer
API: Application programming interface
CAP: Computer-assisted pathology
CC: Creative Commons
Curie: Institut Curie
CWRUC: Case Western Reserve University
DL: Deep learning
DS1/DS2: Dataset 1/Dataset 2
EULA: End User License Agreement
FOV: Field of view
GBM: Glioblastoma multiforme tumor
H&E: Hematoxylin & eosin
HER2: Human epidermal growth factor receptor 2
HNSCC: Head and neck squamous cell carcinoma
IHC: Immunohistochemistry
IITG: Indian Institute of Technology Guwahati
JB: Jules Bordet Institute
LDA: Latent Dirichlet Allocation
LGG: Lower grade glioma tumor
NSLC: Non-small cell lung cancer
ROI: Region of interest
RUMC: Radboud University Medical Center
SHSC: Sunnybrook Health Sciences Centre
sTIL: Stromal tumor-infiltrating lymphocyte
TCGA: The Cancer Genome Atlas Program
TCIA: The Cancer Imaging Archive
TIL: Tumor-infiltrating lymphocyte
TIL-WG: TIL working group
TNBC: Triple-negative breast cancer
UHCW: University Hospitals Coventry and Warwickshire
VTA: Visual tumor-infiltrating lymphocyte assessment
WSI: Whole slide image
